# Supplementary material for: Metapangenomics of the oral microbiome provides insights into habitat adaptation and cultivar diversity
Source: Genome Biol. 2020 Dec 16;21:293. doi: 10.1186/s13059-020-02200-2 (PMC7739467; doi:10.1186/s13059-020-02200-2)
Supplement: Supplementary file 1 — Additional file 1. Supplementary text and Figures. [file 13059_2020_2200_MOESM1_ESM.pdf]

Supplementary Text

Detecting and defining homology in a pangenome

A crucial element underlying construction of a pangenome is being able to identify and group homologous genes. Within a group of closely related genomes, amino acid sequences of homologous genes are likely to be largely conserved across genomes while non-homologous genes both within and across genomes are distinct. Thus, clusters for a species- or genus-level pangenome may be unambiguous. Nonetheless, ambiguous homology and errors in clustering may occur. We used two methods to investigate the overall robustness and validity of our homology definitions – first, determining the robustness of the pangenome to various amino acid similarity thresholds, and second, assessing the level of functional heterogeneity within our gene clusters.

The pangenome construction approach we used compares amino acid sequences for all gene pairs, prunes weak hits, and resolves the network of hits with the Markov Cluster Algorithm (MCL) to determine gene clusters (Delmont & Eren 2018; http://www.merenlab.org/p). MCL uses a hyperparameter, "inflation," to adjust the clustering sensitivity, i.e., the tendency to split clusters. To gauge robustness of the pangenome to the inflation parameter of the MCL algorithm, we varied the inflation parameters by ± 2. The resultant number of gene clusters was quantitatively similar (Table S1), differing by <0.5% for *H. parainfluenzae* and <2.5% for *Rothia*, and the pangenome arrangement was qualitatively similar in that the overall pattern and relative size of the genus core (in the case of *Rothia*), species cores, and accessory genome remained nearly identical (Additional file 1: Fig. S7).

Gene clusters are defined purely by amino acid sequence similarity. Although functional similarity is not part of the definition, nevertheless, intuitively one expects to produce gene clusters that are composed of genes with similar function. We assessed the validity of this expectation by assessing the fraction of gene clusters whose constituent genes were annotated with different COG functions. Heterogeneity of functional annotation within a gene cluster was rare in our data; for *H. parainfluenzae*, only 2.6% (75 out of 2892 gene clusters with predicted

COG functions) of gene clusters had within-cluster functional heterogeneity, and *Rothia* was comparably low at 3.5% (96 of 2757 gene clusters with COG annotation).

For the specific gene clusters that we focused on in this manuscript, we used two additional tests to assess the internal consistency of the gene clusters – functional annotation and manually inspection of sequence alignments.

Gene clusters may be legitimately split according to amino acid sequence, and yet still represent homologous genes carrying out the same function. For gene clusters identified as unique to a group of interest, our functional annotation test consisted of comparing the predicted function of that gene cluster to functions of gene clusters characteristic of other groups. For example, of the gene clusters that were shared exclusively to *Rothia* sp. strains E04 and C03 (the two genomes enriched in buccal mucosa metagenomes), three had functional annotation. However, other gene clusters with identical predicted functions were found in other *Rothia* genomes; therefore we did not hypothesize that these three gene clusters conferred functions potentially important for differential survival in the buccal mucosa environment. The sequence divergence within those gene clusters may confer differential fitness between habitats, however, we do not feel confident enough to put forward that hypothesis given this data.

Additionally, the internal consistency of a gene cluster can be investigated by inspecting the alignment of its constituent amino acid sequences. An alignment of homologs should produce clearly conserved regions across the majority of the sequence with few gaps. For instance, in our investigation of the *Haemophilus parainfluenzae*, we noticed that the TD-abundant strains were characterized by three gene clusters encoding the three subunits of oxaloacetate dehydrogenase. A single non-TD strain (*Haemophilus parainfluenzae* C2004002729) also contained one of the three gene clusters. By inspecting the sequence alignment, which can be obtained from the aa_sequence column of Additional file 3 by searching for “oadA” or “GC_00001928” in Additional file 3, we discovered that the sequence from the non-TD genome

was aberrant relative to the other sequences with numerous gaps and many mismatches. Based on the poor alignment, the inclusion of this non-TD gene sequence in the gene cluster likely reflects mis-assignment to this gene cluster. We therefore consider the oxaloacetate decarboxylase operon as exclusive to the genomes of TD-abundant strains.

Functions of the core and accessory genome for **H. parainfluenzae** *and* **Rothia**

In addition to comparing differences between genomes based on gene content, we also investigated functional differences between core and accessory genes and between species of *Rothia* and strains of *H. parainfluenzae*.

To investigate functional similarities and differences between core and accessory genes, we assessed the frequencies of each COG category in core, singleton accessory, and intermediate accessory genes as identified based on the pangenome. For simplicity we compared only genes assigned an single COG category and omitted genes that were assigned multiple COG categories. For *H. parainfluenzae*, the core consisted of gene clusters shared by all 33 genomes; the singleton accessory genome, gene clusters found in exactly one genome; and the intermediate accessory genome, gene clusters occurring in 2-32 genomes. Overall, each portion of the pangenome contained genes belonging to each COG category (Additional file 1: Fig. S3A) but the frequencies differed. For example, genes involved in translation (J) and nucleotide metabolism (F) were both more numerous and proportionally more enriched in the core genome. On the other hand, defense mechanisms (V) and the mobilome (X) were more abundant in both the singleton and the intermediate accessory genome.

To investigate functional enrichment in one set of genomes compared to another, we recorded the proportion of genomes containing each TIGRFAM function. From this proportional data, the enrichment of each function in each group was determined using a logistic regression by the method of Shaiber et al. (2020). The full enrichment data is presented in Additional file 4 for each gene. To obtain a high-level view of which group(s) were more similar based on shared functions, we aggregated the enrichment scores by subtracting the mean proportional occurrence of each function in the group(s) in which it was not enriched from the mean of its proportional occurrence in the group(s) in which the TIGRFAM was enriched (Additional file 1: Fig. S3B). For example, if a function was enriched in Groups 1 and 2 with a proportional occurrence of 1 and 0.8 in Groups 1 and 2 but also 0.1 in Group 3, the aggregate

enrichment would be (0.8 + 1)/2 – 0.1 = 0.8. This aggregate enrichment of each function is shown in Additional file 1: Fig. S3B. The three genes of the oxaloacetate operon unique to Group 2 stand out clearly, but more broadly the functional similarity between groups can be estimated. Group 2 and Group 3 share more genes with higher enrichment than do Group 1 and Group 2, or Group 1 and Group 3. This observation agrees with the arrangement of genomes based on gene cluster content shown as the dendrogram arranging genome layers in Figure 2, which places Group 2 sister to Group 3.

Functional enrichment analysis indicated that *Rothia* species with similar gene cluster content also contained similar functions. Predicted TIGRFAM functions were used to apply the same functional enrichment analysis as for *H. parainfluenzae*, but this time the groups were the three *Rothia* species (Additional file 1: Fig. S3C, Additional file 6). Unlike the *H. parainfluenzae* analysis, the number of genomes per group varied much more substantially, with 48 *R. mucilaginosa*, 15 *R. dentocariosa*, and 4 *R. aeria* genomes. Yet, *R. dentocariosa* and *R. aeria* were still more functionally similar than either were to *R. mucilaginosa* based on aggregate enrichment scores (Additional file 1: Fig. S3C), agreeing with the similarity of *R. dentocariosa* and *R. aeria* genomes based on gene cluster content (Figure 3 dendrogram).

The functions enriched in each species also revealed possible sources of niche differentiation. Two functions were found in all 15 *R. dentocariosa* genomes but no other *Rothia* species, a PTS-system sucrose transporter component and a transcription repressor gene (Additional file 6). Further, of the 13 functions core to all *R. dentocariosa* and *R. aeria* genomes but absent from all *R. mucilaginosa* genomes, three were cytochrome related (Additional file 6). As both *R. dentocariosa* and *R. aeria* appear most abundant in plaque (Figure 3 heatmap), these cytochrome differences relative to *R. mucilaginosa* could potentially reflect selection by the different oxygen conditions of their respective microhabitats within tongue and plaque habitats.

Supplementary Figure Legends

Fig. S1. Flowchart of key methods and bioinformatic analyses performed. Boxes represent datasets (color coded by category / filetype), and arrows show the programs used to connect or transform the data. The shaded portion on the right (starting with "Individual metagenomes") was performed for each oral site independently (i.e. once each for tongue dorsum, buccal mucosa, and supragingival plaque), and then the habitat-specific metapangenomes were combined onto a single pangenome, as described in the Methods.

Fig. S2. Gene detection in metagenomes is largely bimodal. For all metagenomes covering at least half the nucleotides in a genome, the detection (fraction of each gene receiving any coverage at all) of all genes that genome was counted. For **A)** *H. parainfluenzae* and **B)** *Rothia* spp., the number of metagenomes (y axis) providing each observed gene detection is plotted as a histogram. The genes were split into two categories (colors) – those determined to be environmentally accessory genes (EAG) or environmentally core genes (ECG) by having a median coverage less than or at least 0.25x the parent genome's median coverage, respectively. **C)** and **D)** show the probability density function for *H. parainfluenzae* and *Rothia,* respectively, using the same gene detection data shown in **A** and **B**. Detection is shown on the x-axis, and the y-axis shows the probability of the metagenomes producing that detection. The distribution of detections for EAGs are shown in orange and ECG in blue.

Fig. S3. Functional similarities in the pangenome. A) COG categories of the different *H. parainfluenzae* pangenome fractions (x-axis). The pangenome was apportioned into the core genome (gene clusters found in all genomes), the singleton accessory genome (gene clusters in exactly one genome), and the intermediate accessory genome (the remaining gene clusters). The height of each bar shows the number of COG-annotated gene clusters per pangenome portion, colored by COG category. Only gene clusters annotated with a single COG category, or none, were included. **B)** Enrichment of TIGRFAM functions in by group of *H. parainfluenzae* genomes detected in the pangenome (Figure 2). Each group or combination of groups is listed along the x axis. The y-axis is the count of TIGRFAM genes enriched by group, with each gene colored by its aggregate proportional enrichment. Aggregate enrichment was calculated for each TIGRFAM by subtracting the mean proportional occurrence of each function in the group(s) in which it was not enriched from the mean of its proportional occurrence in the group(s) in which the TIGRFAM was enriched. **C)** The same analysis as in **B** is shown but for species of the genus *Rothia*.

Fig. S4. Comparison of genome relatedness by gene content with phylogenomics, 16S, and sourmash. A) Phylogenomic tree based on 139 concatenated single-copy core genes. Tip names colored in red correspond to those of Group 2 in Figure 2. **B)** Pangenome is arranged as in Figure 2, but the yellow heatmap shows 16S % similarity and the red heatmap shows sourmash similarity. Each heatmap's order from bottom to top matches the order from left to right.

Fig. S5. Syntenic arrangement of *Rothia mucilaginosa* genomes relative to a *R. mucilaginosa* subgroup 1 genome (*R.* sp. E04). Gene clusters from *R. mucilaginosa* genomes are arranged in syntenic order according to *R.* sp. E04 (red arrow); gene clusters not found in *R.* sp. E04 are omitted. Red arrows above and below mark the 22 gene clusters uniquely shared by both R. sp E04 and R. sp C03 (blue arrow). The order and spacing of layers is identical to that Figure 2 but linearized.

Fig. S6. *R.* sp. C03 gene-level recruitment of HMP metagenomes. Layout as in Figure 4A but for *R.* sp. C03. Genes shared with *R.* sp. E04 are marked to also show that the genes unique to the BM-enriched subgroup are also well distributed throughout the genome.

Fig. S7. Comparison of varying MCL inflation factors on pangenome structure. Identical pangenomes were run but with varying MCL inflation factors. The left and right columns of pangenome plots show the *H. parainfluenzae* and *Rothia* pangenomes, respectively. The MCL inflation parameter used for each pangenome shown is listed next to the central dendrogram.

Fig. S1

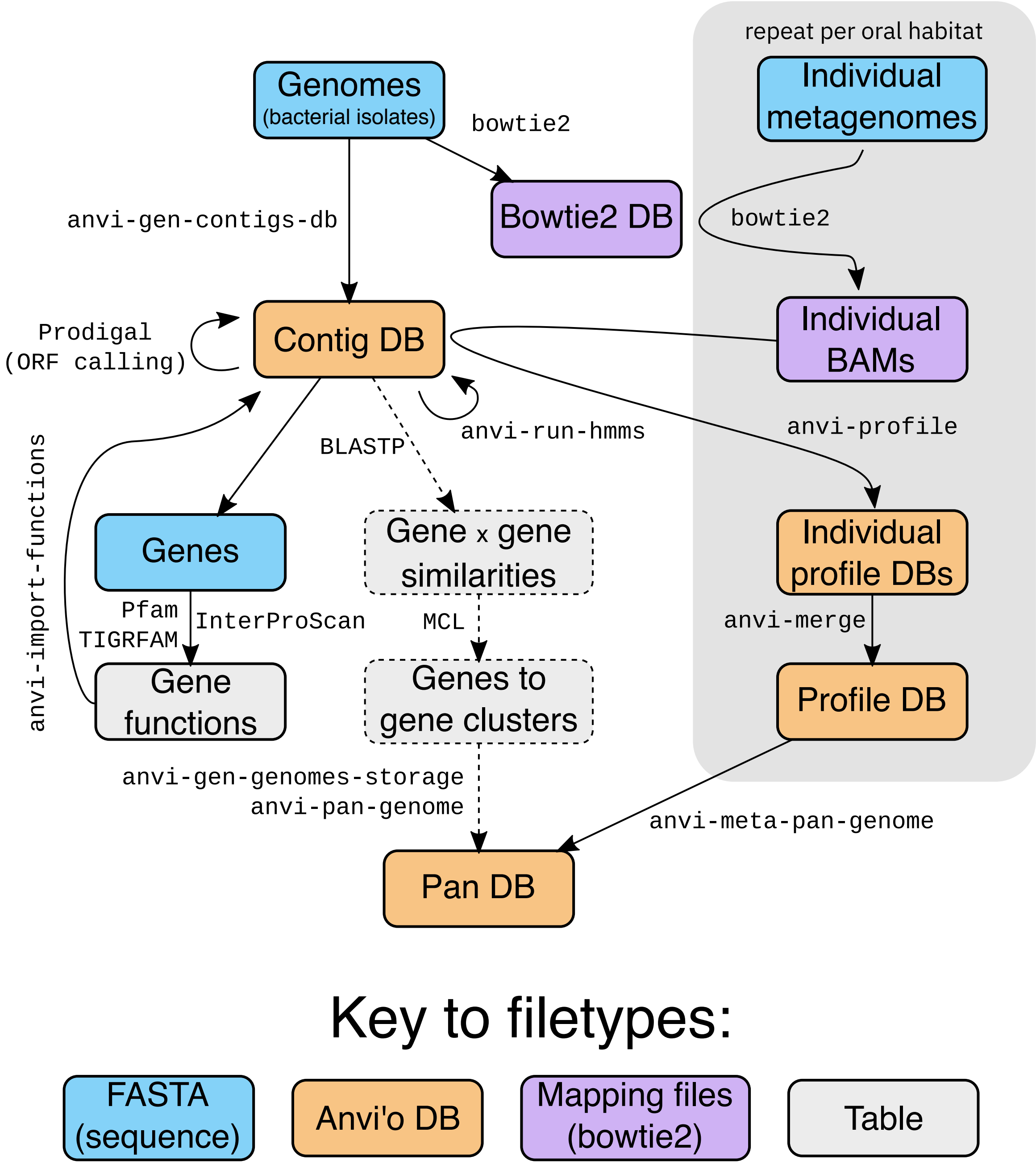

repeat per oral habitat
Genomes
(bacterial isolates)
bowtie2
Individual metagenomes
Bowtie2 DB
anvi-gen-contigs-db
bowtie2
Contig DB
Individual BAMs
Prodigal
(ORF calling)
anvi-run-hmms
anvi-profile
BLASTP
anvi-import-functions
Genes
Gene x gene similarities
Individual profile DBs
Pfam
TIGRFAM
InterProScan
MCL
anvi-merge
Gene functions
Genes to gene clusters
Profile DB
anvi-gen-genomes-storage
anvi-pan-genome
anvi-meta-pan-genome
Pan DB
Key to filetypes:
FASTA (sequence)
Anvi'o DB
Mapping files (bowtie2)
Table

Fig. S2

A

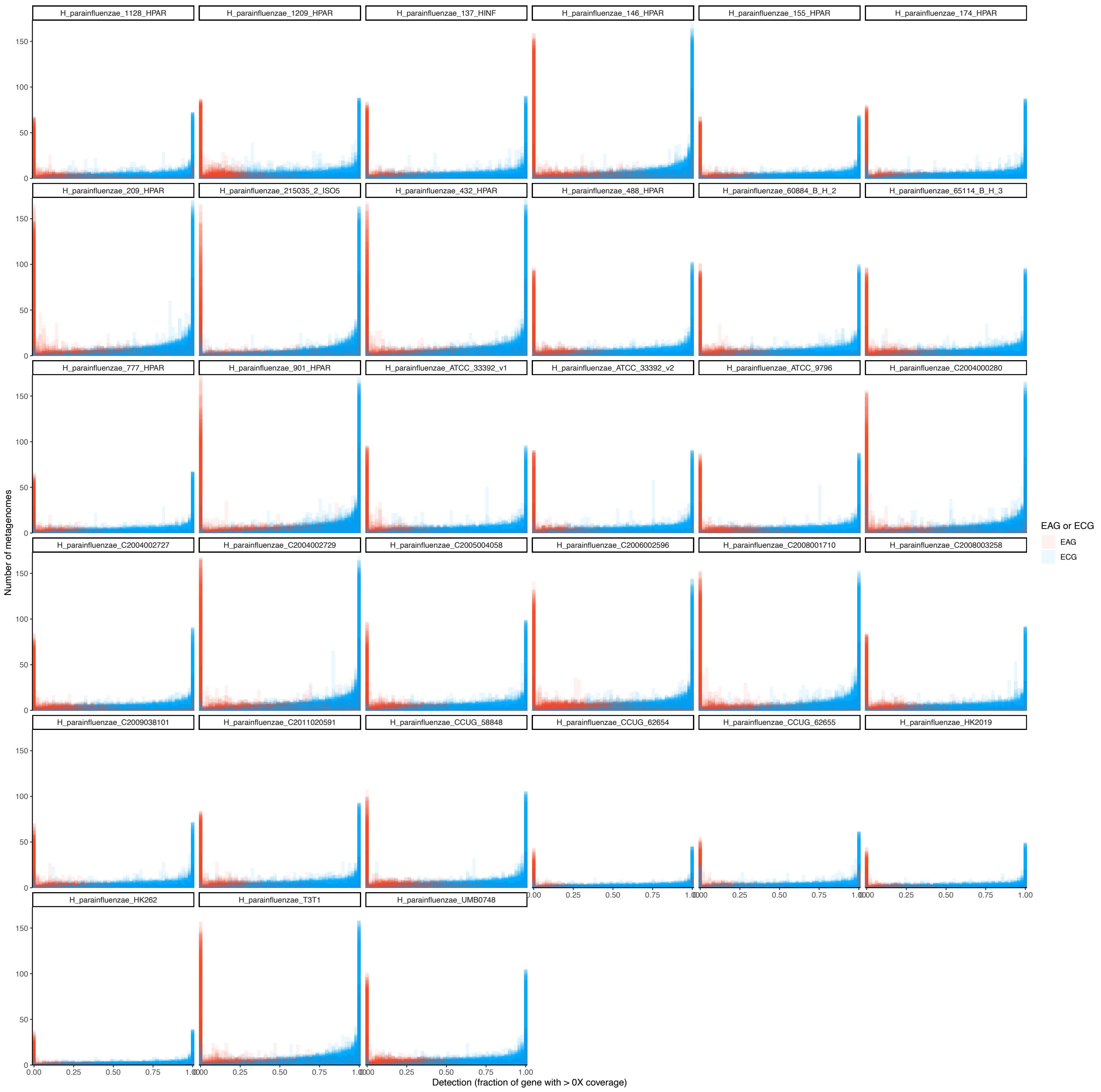

H_parainfluenzae_1128_HPAR
H_parainfluenzae_1209_HPAR
H_parainfluenzae_137_HINF
H_parainfluenzae_146_HPAR
H_parainfluenzae_155_HPAR
H_parainfluenzae_174_HPAR
H_parainfluenzae_209_HPAR
H_parainfluenzae_215035_2_ISO5
H_parainfluenzae_432_HPAR
H_parainfluenzae_488_HPAR
H_parainfluenzae_60884_B_H_2
H_parainfluenzae_65114_B_H_3
H_parainfluenzae_777_HPAR
H_parainfluenzae_901_HPAR
H_parainfluenzae_ATCC_33392_v1
H_parainfluenzae_ATCC_33392_v2
H_parainfluenzae_ATCC_9796
H_parainfluenzae_C2004000280
H_parainfluenzae_C2004002727
H_parainfluenzae_C2004002729
H_parainfluenzae_C2005004058
H_parainfluenzae_C2006002596
H_parainfluenzae_C2008001710
H_parainfluenzae_C2008003258
H_parainfluenzae_C2009038101
H_parainfluenzae_C2011020591
H_parainfluenzae_CCUG_58848
H_parainfluenzae_CCUG_62654
H_parainfluenzae_CCUG_62655
H_parainfluenzae_HK2019
H_parainfluenzae_HK262
H_parainfluenzae_T3T1
H_parainfluenzae_UMB0748
Number of metagenomes
0
50
100
150
0.00
0.25
0.50
0.75
1.00
Detection (fraction of gene with > 0X coverage)
EAG or ECG
EAG
ECG

Fig. S2

B

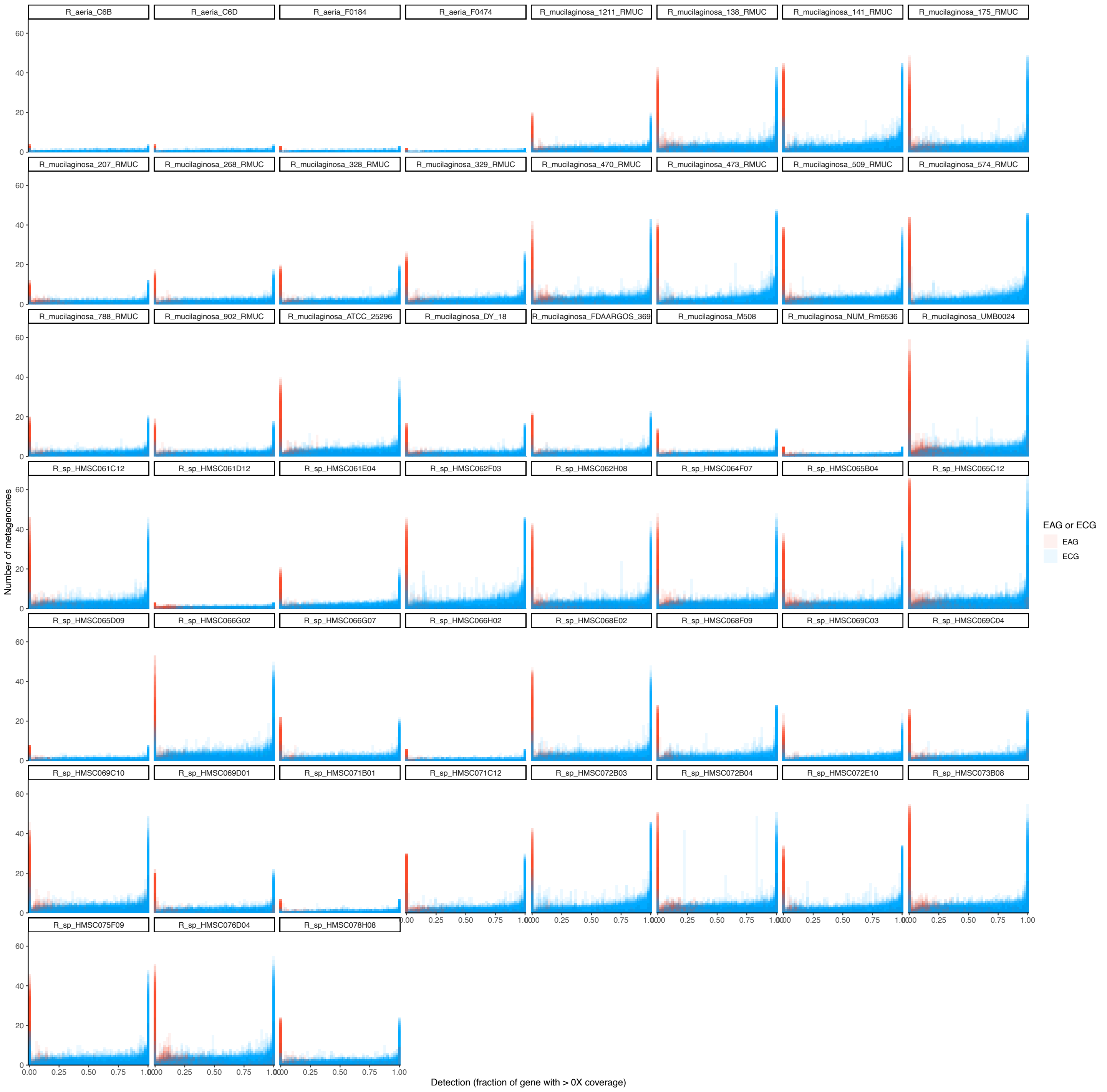

R_aeria_C6B
R_aeria_C6D
R_aeria_F0184
R_aeria_F0474
R_mucilaginosa_1211_RMUC
R_mucilaginosa_138_RMUC
R_mucilaginosa_141_RMUC
R_mucilaginosa_175_RMUC
R_mucilaginosa_207_RMUC
R_mucilaginosa_268_RMUC
R_mucilaginosa_328_RMUC
R_mucilaginosa_329_RMUC
R_mucilaginosa_470_RMUC
R_mucilaginosa_473_RMUC
R_mucilaginosa_509_RMUC
R_mucilaginosa_574_RMUC
R_mucilaginosa_788_RMUC
R_mucilaginosa_902_RMUC
R_mucilaginosa_ATCC_25296
R_mucilaginosa_DY_18
R_mucilaginosa_FDAARGOS_369
R_mucilaginosa_M508
R_mucilaginosa_NUM_Rm6536
R_mucilaginosa_UMB0024
R_sp_HMSC061C12
R_sp_HMSC061D12
R_sp_HMSC061E04
R_sp_HMSC062F03
R_sp_HMSC062H08
R_sp_HMSC064F07
R_sp_HMSC065B04
R_sp_HMSC065C12
R_sp_HMSC065D09
R_sp_HMSC066G02
R_sp_HMSC066G07
R_sp_HMSC066H02
R_sp_HMSC068E02
R_sp_HMSC068F09
R_sp_HMSC069C03
R_sp_HMSC069C04
R_sp_HMSC069C10
R_sp_HMSC069D01
R_sp_HMSC071B01
R_sp_HMSC071C12
R_sp_HMSC072B03
R_sp_HMSC072B04
R_sp_HMSC072E10
R_sp_HMSC073B08
R_sp_HMSC075F09
R_sp_HMSC076D04
R_sp_HMSC078H08
Number of metagenomes
0
20
40
60
0.00
0.25
0.50
0.75
1.00
Detection (fraction of gene with > 0X coverage)
EAG or ECG
EAG
ECG

Fig. S2

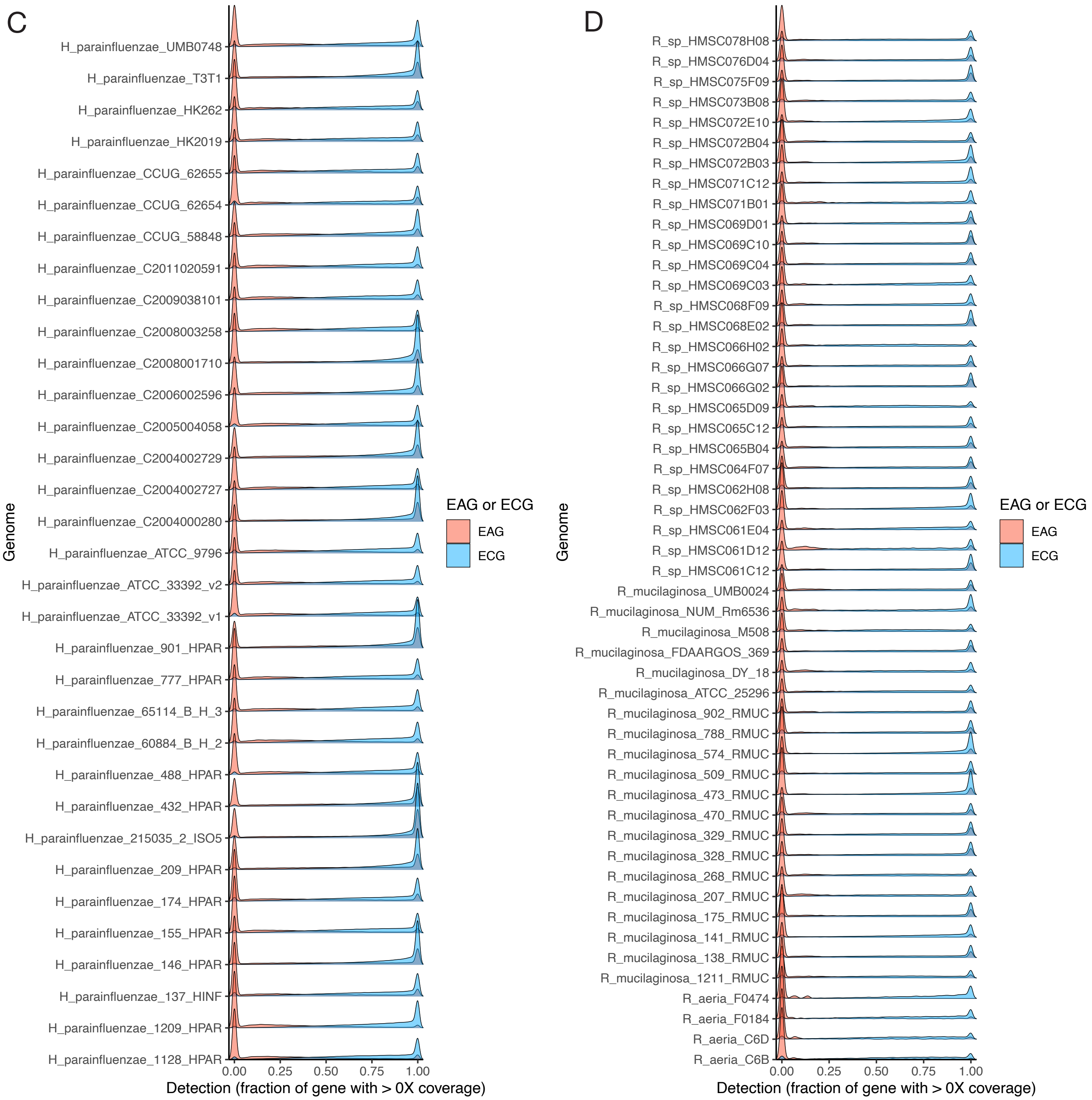
C
H_parainfluenzae_UMB0748
H_parainfluenzae_T3T1
H_parainfluenzae_HK262
H_parainfluenzae_HK2019
H_parainfluenzae_CCUG_62655
H_parainfluenzae_CCUG_62654
H_parainfluenzae_CCUG_58848
H_parainfluenzae_C2011020591
H_parainfluenzae_C2009038101
H_parainfluenzae_C2008003258
H_parainfluenzae_C2008001710
H_parainfluenzae_C2006002596
H_parainfluenzae_C2005004058
H_parainfluenzae_C2004002729
H_parainfluenzae_C2004002727
H_parainfluenzae_C2004000280
H_parainfluenzae_ATCC_9796
H_parainfluenzae_ATCC_33392_v2
H_parainfluenzae_ATCC_33392_v1
H_parainfluenzae_901_HPAR
H_parainfluenzae_777_HPAR
H_parainfluenzae_65114_B_H_3
H_parainfluenzae_60884_B_H_2
H_parainfluenzae_488_HPAR
H_parainfluenzae_432_HPAR
H_parainfluenzae_215035_2_ISO5
H_parainfluenzae_209_HPAR
H_parainfluenzae_174_HPAR
H_parainfluenzae_155_HPAR
H_parainfluenzae_146_HPAR
H_parainfluenzae_137_HINF
H_parainfluenzae_1209_HPAR
H_parainfluenzae_1128_HPAR
Genome
0.00
0.25
0.50
0.75
1.00
Detection (fraction of gene with > 0X coverage)
EAG or ECG
EAG
ECG
D
R_sp_HMSC078H08
R_sp_HMSC076D04
R_sp_HMSC075F09
R_sp_HMSC073B08
R_sp_HMSC072E10
R_sp_HMSC072B04
R_sp_HMSC072B03
R_sp_HMSC071C12
R_sp_HMSC071B01
R_sp_HMSC069D01
R_sp_HMSC069C10
R_sp_HMSC069C04
R_sp_HMSC069C03
R_sp_HMSC068F09
R_sp_HMSC068E02
R_sp_HMSC066H02
R_sp_HMSC066G07
R_sp_HMSC066G02
R_sp_HMSC065D09
R_sp_HMSC065C12
R_sp_HMSC065B04
R_sp_HMSC064F07
R_sp_HMSC062H08
R_sp_HMSC062F03
R_sp_HMSC061E04
R_sp_HMSC061D12
R_sp_HMSC061C12
R_mucilaginosa_UMB0024
R_mucilaginosa_NUM_Rm6536
R_mucilaginosa_M508
R_mucilaginosa_FDAARGOS_369
R_mucilaginosa_DY_18
R_mucilaginosa_ATCC_25296
R_mucilaginosa_902_RMUC
R_mucilaginosa_788_RMUC
R_mucilaginosa_574_RMUC
R_mucilaginosa_509_RMUC
R_mucilaginosa_473_RMUC
R_mucilaginosa_470_RMUC
R_mucilaginosa_329_RMUC
R_mucilaginosa_328_RMUC
R_mucilaginosa_268_RMUC
R_mucilaginosa_207_RMUC
R_mucilaginosa_175_RMUC
R_mucilaginosa_141_RMUC
R_mucilaginosa_138_RMUC
R_mucilaginosa_1211_RMUC
R_aeria_F0474
R_aeria_F0184
R_aeria_C6D
R_aeria_C6B
Genome
0.00
0.25
0.50
0.75
1.00
Detection (fraction of gene with > 0X coverage)
EAG or ECG
EAG
ECG

Fig. S3

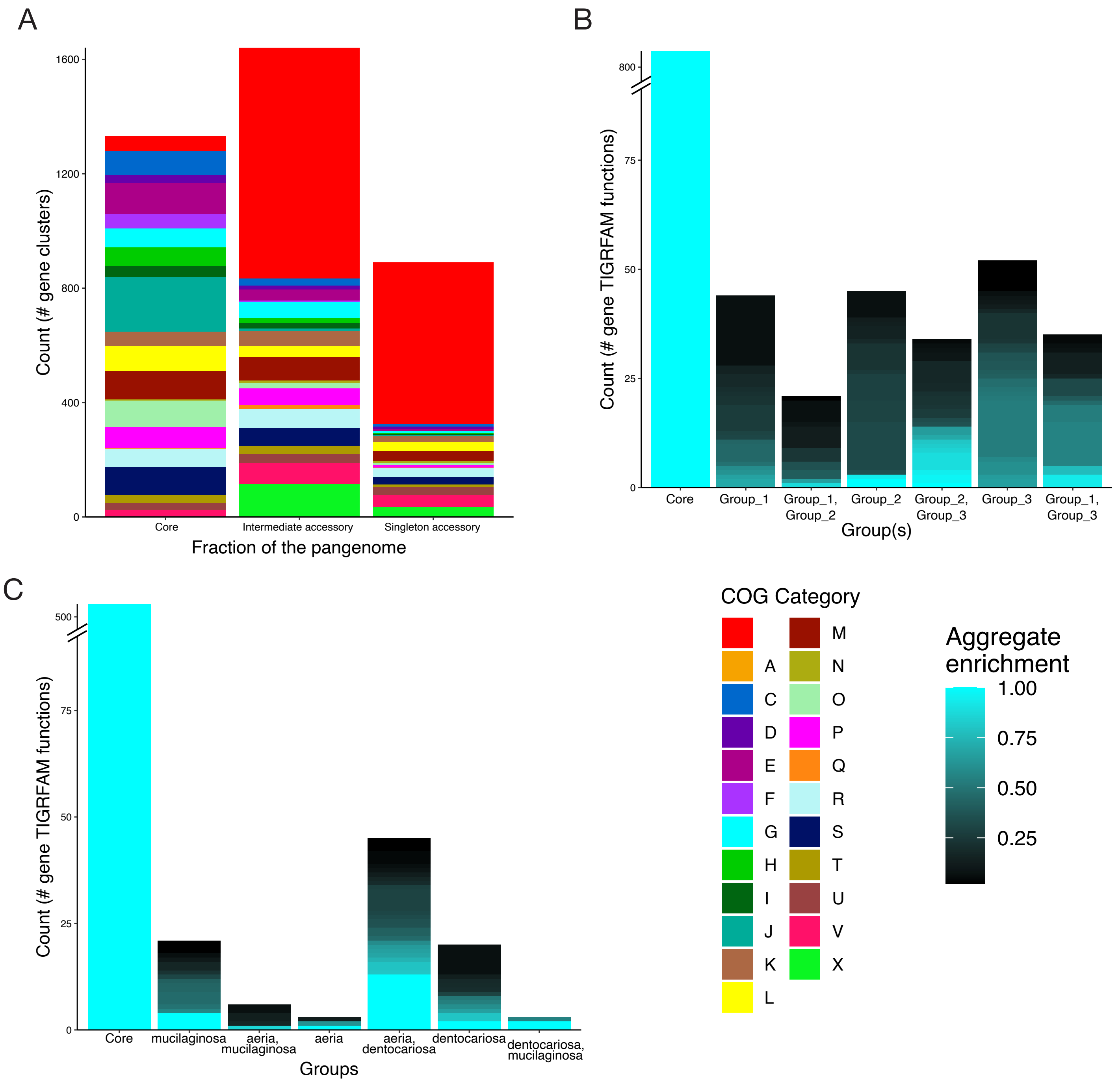
A
1600
1200
800
400
0
Count (# gene clusters)
Core
Intermediate accessory
Singleton accessory
Fraction of the pangenome
B
800
75
50
25
0
Count (# gene TIGRFAM functions)
Core
Group_1
Group_1, Group_2
Group_2
Group_2, Group_3
Group_3
Group_1, Group_3
Group(s)
C
500
75
50
25
0
Count (# gene TIGRFAM functions)
Core
mucilaginosa
aeria, mucilaginosa
aeria
aeria, dentocariosa
dentocariosa
dentocariosa, mucilaginosa
Groups
COG Category
A
C
D
E
F
G
H
I
J
K
L
M
N
O
P
Q
R
S
T
U
V
X
Aggregate enrichment
1.00
0.75
0.50
0.25

Fig. S4

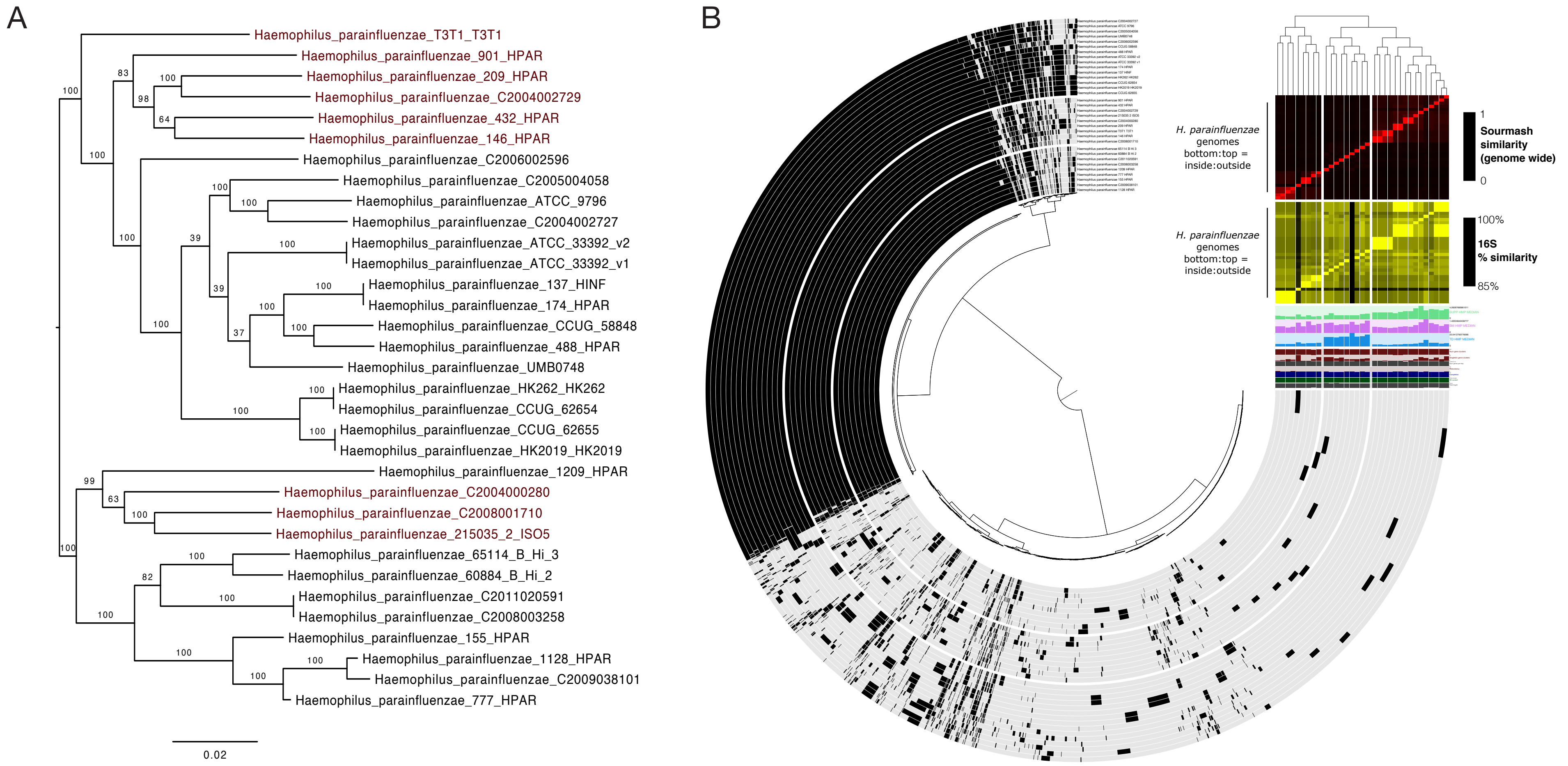

A
Haemophilus_parainfluenzae_T3T1_T3T1
Haemophilus_parainfluenzae_901_HPAR
Haemophilus_parainfluenzae_209_HPAR
Haemophilus_parainfluenzae_C2004002729
Haemophilus_parainfluenzae_432_HPAR
Haemophilus_parainfluenzae_146_HPAR
Haemophilus_parainfluenzae_C2006002596
Haemophilus_parainfluenzae_C2005004058
Haemophilus_parainfluenzae_ATCC_9796
Haemophilus_parainfluenzae_C2004002727
Haemophilus_parainfluenzae_ATCC_33392_v2
Haemophilus_parainfluenzae_ATCC_33392_v1
Haemophilus_parainfluenzae_137_HINF
Haemophilus_parainfluenzae_174_HPAR
Haemophilus_parainfluenzae_CCUG_58848
Haemophilus_parainfluenzae_488_HPAR
Haemophilus_parainfluenzae_UMB0748
Haemophilus_parainfluenzae_HK262_HK262
Haemophilus_parainfluenzae_CCUG_62654
Haemophilus_parainfluenzae_CCUG_62655
Haemophilus_parainfluenzae_HK2019_HK2019
Haemophilus_parainfluenzae_1209_HPAR
Haemophilus_parainfluenzae_C2004000280
Haemophilus_parainfluenzae_C2008001710
Haemophilus_parainfluenzae_215035_2_ISO5
Haemophilus_parainfluenzae_65114_B_Hi_3
Haemophilus_parainfluenzae_60884_B_Hi_2
Haemophilus_parainfluenzae_C2011020591
Haemophilus_parainfluenzae_C2008003258
Haemophilus_parainfluenzae_155_HPAR
Haemophilus_parainfluenzae_1128_HPAR
Haemophilus_parainfluenzae_C2009038101
Haemophilus_parainfluenzae_777_HPAR
0.02
B
H. parainfluenzae genomes bottom:top = inside:outside
1
Sourmash similarity (genome wide)
0
H. parainfluenzae genomes bottom:top = inside:outside
100%
16S % similarity
85%

Fig. S5

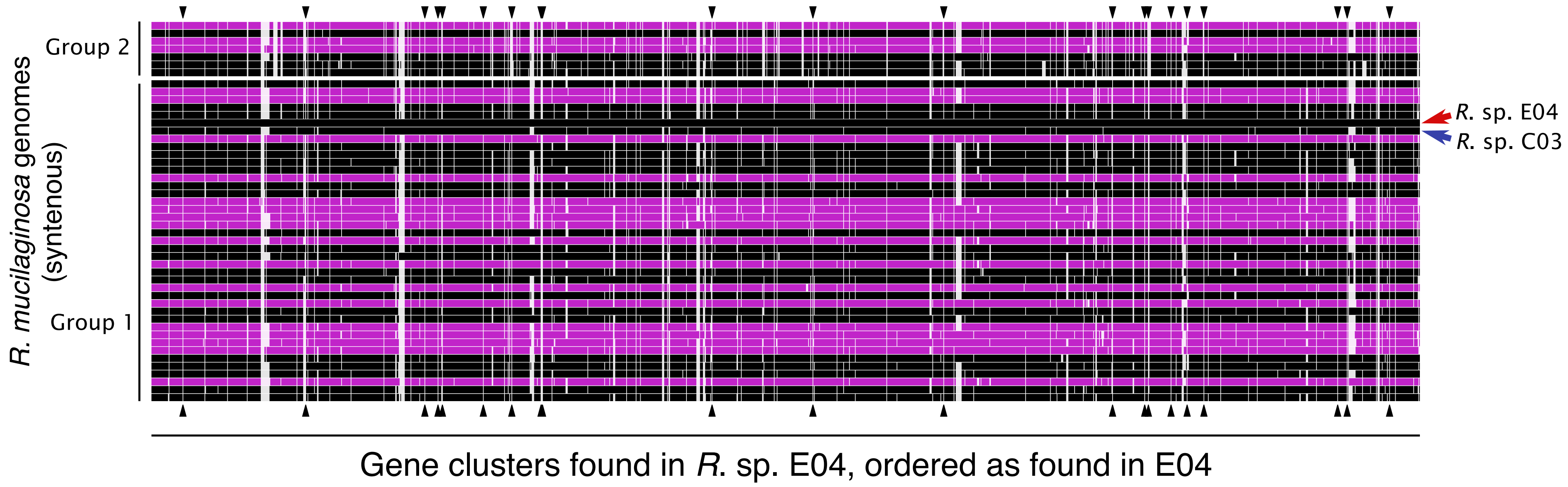

Group 2
Group 1
R. mucilaginosa genomes (syntenous)
R. sp. E04
R. sp. C03
Gene clusters found in *R.* sp. E04, ordered as found in E04

Fig. S6

Rothia sp HMSC069C03

Items order: <> User order (D: none; L: none) | Current view: single | Samples order: custom

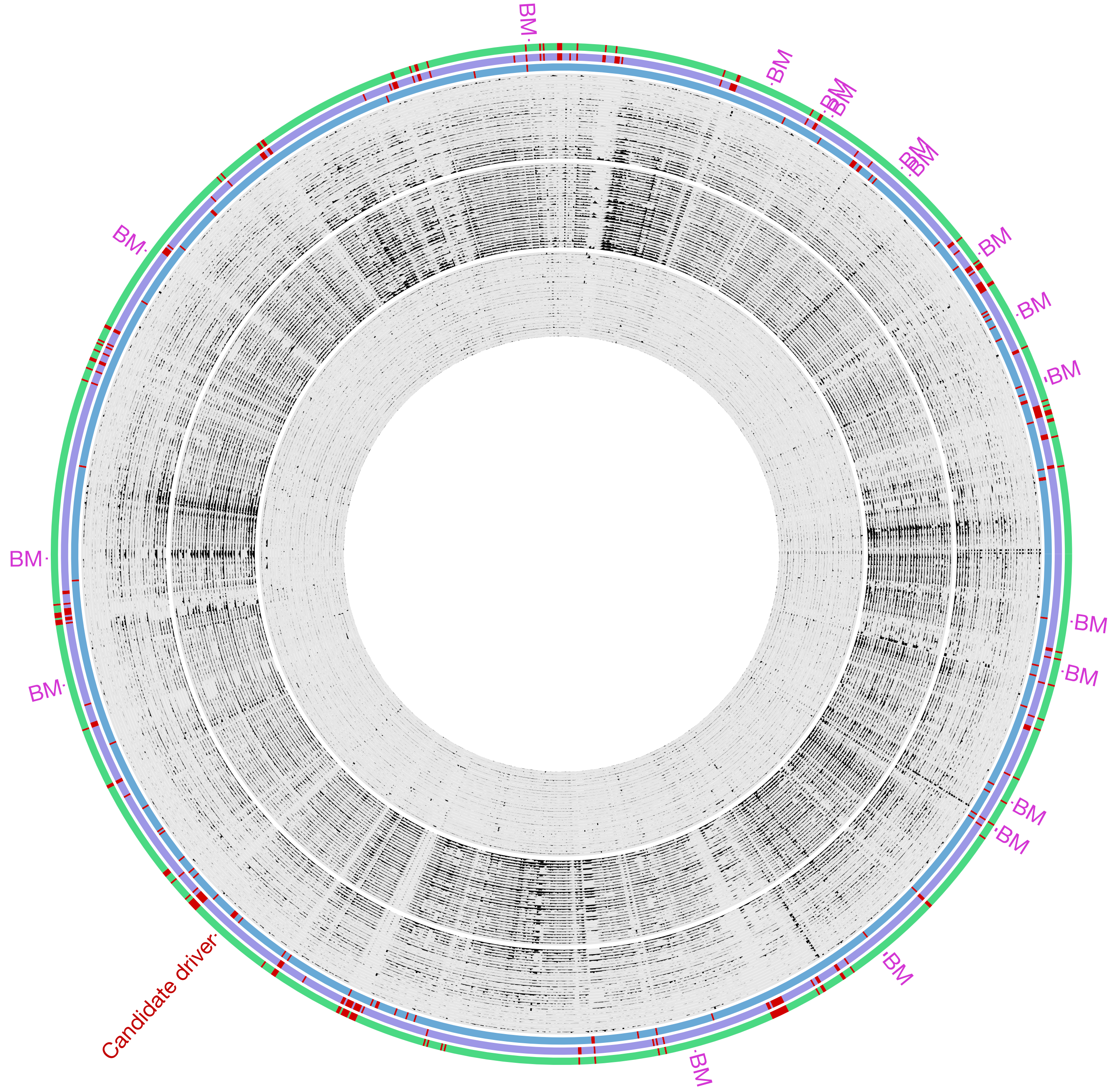

TD detection DETECTED (1721) NOT DETECTED (69)

BM detection DETECTED (1633) NOT DETECTED (157)

SUPP detection DETECTED (1667) NOT DETECTED (123)

Fig. S7

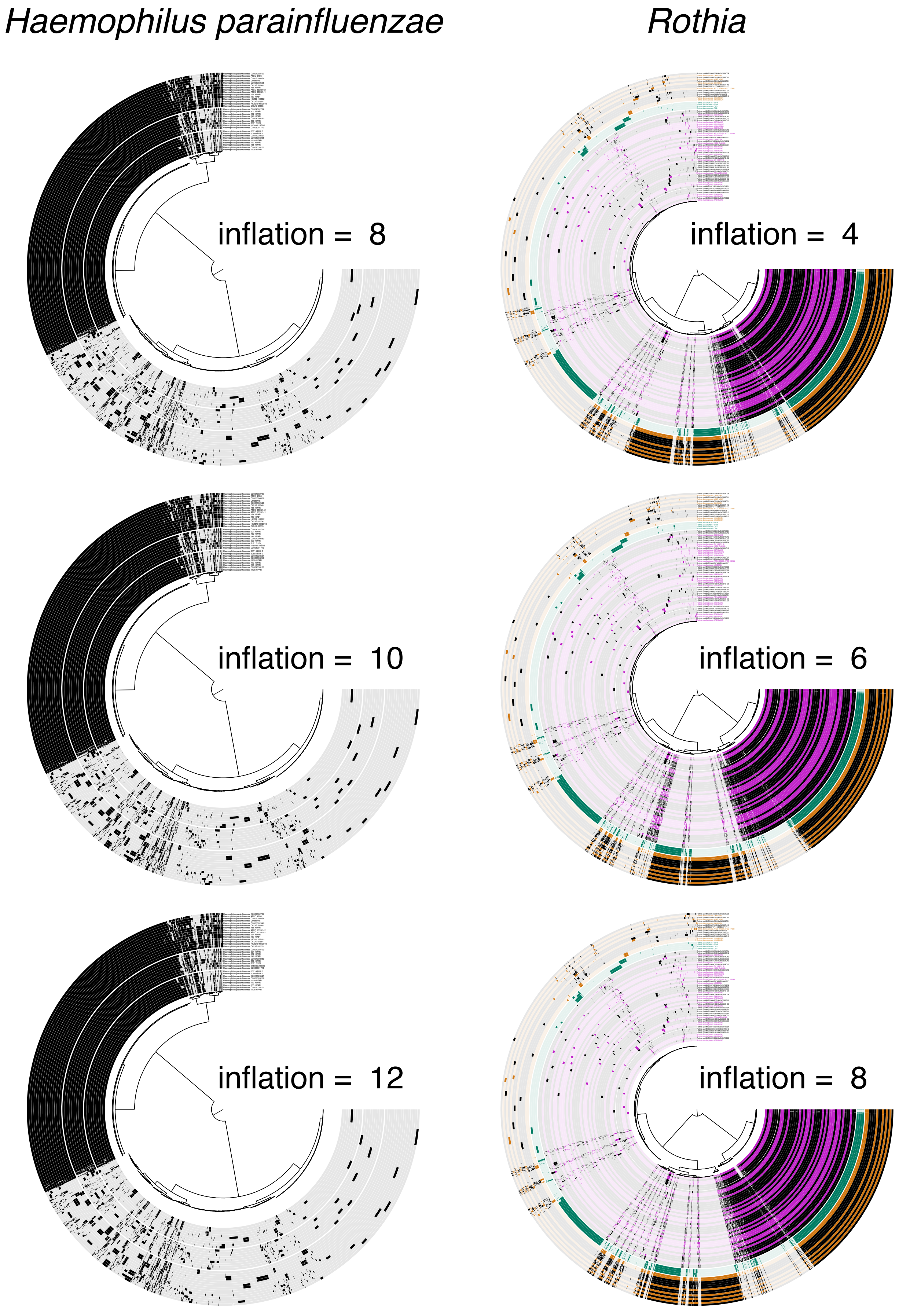

Haemophilus parainfluenzae
Rothia
inflation = 8
inflation = 4
inflation = 10
inflation = 6
inflation = 12
inflation = 8
