# Supplementary material for: Metapangenomics of the oral microbiome provides insights into habitat adaptation and cultivar diversity
Source: Genome Biol. 2020 Dec 16;21:293. doi: 10.1186/s13059-020-02200-2 (PMC7739467; doi:10.1186/s13059-020-02200-2)
Supplement: Supplementary file 8 — Additional file 8: Table S1. Number of gene clusters per pangenome with varying MCL inflation factors. [file 13059_2020_2200_MOESM8_ESM.docx]

**Table S1: Number of gene clusters per pangenome with varying MCL inflation factors.**

|  | ***Rothia* genus** | | | ***H. parainfluenzae*** | | |
| --- | --- | --- | --- | --- | --- | --- |
| **MCL Inflation factor** | 4 | 6* | 8 | 8 | 10* | 12 |
| **Num. gene clusters** | 5,855 | 5,992 | 6,090 | 4,303 | 4,318 | 4,338 |
| **% change from *** | -2.29% | 0% | 1.64% | 0.35% | 0% | 0.46% |

* MCL inflation factor selected for use in all subsequent analyses presented
